# Adaptive Biomarker‐Based Design for Early Phase Clinical Trials

**DOI:** 10.1002/sim.70275

**Published:** 2025-10-09

**Authors:** Alessandra Serra, Gaëlle Saint‐Hilary, Sandrine Guilleminot, Julia Geronimi, Pavel Mozgunov

**Affiliations:** ^1^ University of Cambridge MRC Biostatistics Unit Cambridge UK; ^2^ Department of Statistical Methodology Saryga France; ^3^ Translational Statistics Department Institut de Recherches Internationales Servier Gif‐sur‐Yvette France

**Keywords:** continuous biomarker, early phase, personalized medicine, predictive

## Abstract

Identifying and quantifying predictive biomarkers is a critical issue of Precision Medicine approaches and patient‐centric clinical development strategies. Early phase adaptive designs can improve trial efficiency by allowing for adaptations during the course of the trial. In this work, we are interested in adaptations based on interim analysis permitting a refinement of the existing study population according to their predictive biomarkers. At an early stage, the goal is not to precisely define the target population, but to not miss an efficacy signal that might be limited to a biomarker subgroup. In this work, we propose a one‐arm two‐stage early phase biomarker‐guided design in the setting of an oncology trial where at the time of the interim analysis, several decisions can be made regarding stopping the entire trial early or continuing to recruit patients from the full or a selected patient population. Via simulations, we show that, although the sample size is limited, the proposed design leads to better decision‐making compared to a classical design that does not consider an enrichment expansion.

**Abbreviations**
BMK: biomarker
FA: final analysis
IA: interim analysis
LRV: lower reference value
PoC: proof of concept
TV: target value

## 1 Introduction

Identifying and quantifying predictive biomarkers is a critical issue of Precision Medicine approaches and patient‐centric clinical development strategies. Several clinical trial designs have been proposed for testing the clinical validity of a biomarker and enrichment strategies in late stages of drug development have been widely studied in the literature. An extensive methodological review of biomarker‐guided adaptive designs for Phase II and III of clinical development has been proposed by Antoniou et al. [1] and Superchi et al. [2]. However, the implementation of these strategies in early stages presents significant challenges due to the small sample size and the numerous uncertainties that can arise at this point in the development process. Uncertainties occur because we are often learning about the biomarker at the same time as learning about the treatment. These uncertainties [3] include, for example, the predictive value of the biomarker, the cutoff value of the biomarker used to identify patients in the biomarker‐positive subgroup, the proportion of patients in the biomarker‐positive subgroup and the magnitude of the treatment effect in biomarker‐positive and biomarker‐negative patients. Changes in validation status of the biomarker testing during the trial can also increase complexity.

It is essential that clinical trials assessing predictive biomarkers are properly designed, with the possibility of identifying and confirming the efficacy signal either in the whole patient population, or in a subgroup defined by a biomarker. Two‐arm controlled trials are required to claim that the biomarker affects the effect of the treatment on the outcome and thus to declare its predictiveness and to distinguish it from the prognostic effect [4]. However, early in the development, it might not be possible, despite the hypothesis that the biomarker effect can be of interest. Thus, in cases where there is strong biological rationale and preclinical evidence that a treatment may provide differential benefit in a biomarker‐defined subset, early‐phase Proof of Concept (PoC) studies could be designed to specifically address biomarker‐related questions to improve development efficiency.

Early phase adaptive designs can improve trial efficiency by allowing for adaptions during the course of the trial. Adaptations can include for example early stopping, where futile treatments can be dropped or where efficacious treatments can be declared and selected before the end of the trial [5, 6]. In later stages of drug development, adaptations can also refer to enrichment strategies, where data collected during the interim analyses can inform decisions on whether it is more appropriate to continue the trial with the full population or restrict recruitment to a subset of patients who do respond particularly well to the new treatment [7]. In this work, we are interested in adaptations based on an interim analysis that permits a refinement of the existing study population according to their predictive biomarkers. At an early stage, such as the first PoC study, the goal is not to precisely define the target population, but to detect an efficacy signal that might be limited to a biomarker subgroup. The enrichment strategy can increase the chances of success of the study by allowing its expansion in a restricted population [8].

Single‐arm PoC clinical studies are widely used to accelerate the signal‐finding process in oncology drug development before or in lieu of randomized PoC studies [9]. In this work, we propose a one‐arm two‐stage early phase biomarker‐guided design inspired by the work done by Brannath et al. [10]. The design can assess the probability of reaching success at the end of the trial in the entire or identified subpopulation using information from the first stage of the study to make a decision about stopping early the entire trial or continuing recruitment from the full or the restricted (accordingly to a pre‐specified criterion) patient population. Recruitment might be restricted using a preliminary threshold or cutoff of the biomarker, which is determined at the end of the first stage and divides patients into two subgroups based on the estimated probability of response to treatment. At the end of the second stage, a final decision on whether or not to pursue the development of the experimental treatment is made. The proposed design is compared to a classical design that does not consider an enrichment expansion. We will show that the proposed design leads to better decision‐making compared to the classical one as it results in a higher probability of success at the end of the trial and it leads to a limited proportion of false enrichment.

## 2 Motivating Trial

This work is motivated by a PhI‐II PoC trial in oncology in Servier. A total of 27 patients was initially planned to be recruited for this phase and an interim analysis is planned after the first 14 patients have been treated. Recruited patients are monitored and a continuous biological marker measurement is planned to be collected at baseline for each patient. Some preliminary analytical validations of the bioassay are used to measure the biomarker of interest and this has no prognostic value.

The primary outcome of the trial is the overall response rate and it is assumed that patients with larger values of a biomarker have higher response rates. Then, the population for which the value of the biomarker is above the identified value is referred to as a biomarker‐positive (BMK+) subgroup. The biomarker is supposed to follow a continuous normal distribution.

The objective of the trial was to establish whether the drug was worth pursuing further in the development. Only a futility stop was planned at the time of the interim analysis and the decision was based on the predictive probability of success at the final analysis, based on the data accumulated so far in the full population. In case this probability was found to be sufficiently large, then the study would have continued to the second stage recruiting patients from the overall population. Otherwise, the study would have stopped early.

The objective of this work is to propose a novel design which can incorporate decisions at the interim analysis on whether the drug is worth pursuing further only in the target biomarker‐defined subgroup instead of the overall population. This is of interest because, although not every patient is expected to respond, the characteristics of the groups (in terms of biomarker values) that will respond are not clear and should be defined as part of the trial. Indeed, stopping the trial without restricting the results to a sub‐population might lead to a premature decision.

## 3 Background

In this section, we describe the original design of the motivating trial. The proposed adaptive design in the setting of the motivating example is introduced in Section 4.

### 3.1 Setting

Consider a clinical trial with an active treatment arm. Assume that a patient's outcome, a response rate, follows a binary distribution, thus Y∼Bin(p) with probability of response p. An interim analysis (IA) is performed when $n_{f}$ patients (the subscript f refers to the full population) have been allocated to the experimental arm. The final analysis (FA) is conducted with a total sample size of $N_{f}$ patients. Let $r_{n_{f}}$ be the number of responses after observing $n_{f}$ patients. Let $r_{N_{f}-n_{f}}$ be the number of responses in the second stage in the full population. Let denote by $D_{i}$ the accumulated data up to i patients. Let us assume to measure a biomarker X that follows a continuous normal distribution with $X∼N(\mu_{X},\sigma_{X}^{2})$.

A Bayesian approach to inference is considered in this work. This consists on a probability density function, that is $f_{Y}(p)$ and a prior distribution π(p). Then the posterior distribution $\pi (p\;|\;D_{i})$ is obtained after observing the accumulated data $D_{i}$.

### 3.2 Original Design of the Motivating Trial

Firstly, the decision rules at the end of the trial will be described and then the possible decisions that can be made at the interim analysis will be presented.

The success criteria at the final analysis is defined as the probability that the response rate is greater than a pre‐specified minimal value of accepted efficacy, called Lower Reference Value (LRV), exceeds a pre‐defined level $\alpha_{LRV}$. If instead the probability that the response rate is below a certain desired level of efficacy, called Target (TV), is less than a pre‐defined level $\alpha_{TV}$, then the development process of the drug is stopped. The decision criteria are computed considering the posterior distribution of a beta‐binomial model, with a prior p∼Beta(.5,.5), after observing i patients which is $p\;|\;D_{i}∼Beta(0.5+r_{i},0.5+i-r_{i}).$

The posterior distribution is then compared to pre‐specified thresholds ($\alpha_{TV}$ and $\alpha_{LRV}$) in order to give a recommendation for the next phase of the drug development. Thus, the clinical decisions at the end of the final analysis follow the decision‐making approach proposed by Frewer et al. [11] and can be summarised as:

- if $1-P(p<TV\;|\;D_{i})\leq \alpha_{TV}$, then stop the development (No Go)
- if $1-P(p<LRV\;|\;D_{i})\geq \alpha_{LRV}$, then continue the development (Go)
- none of the above is satisfied then consider (Consider)

where $i=N_{f}$ for the original design.

**FIGURE 1:**
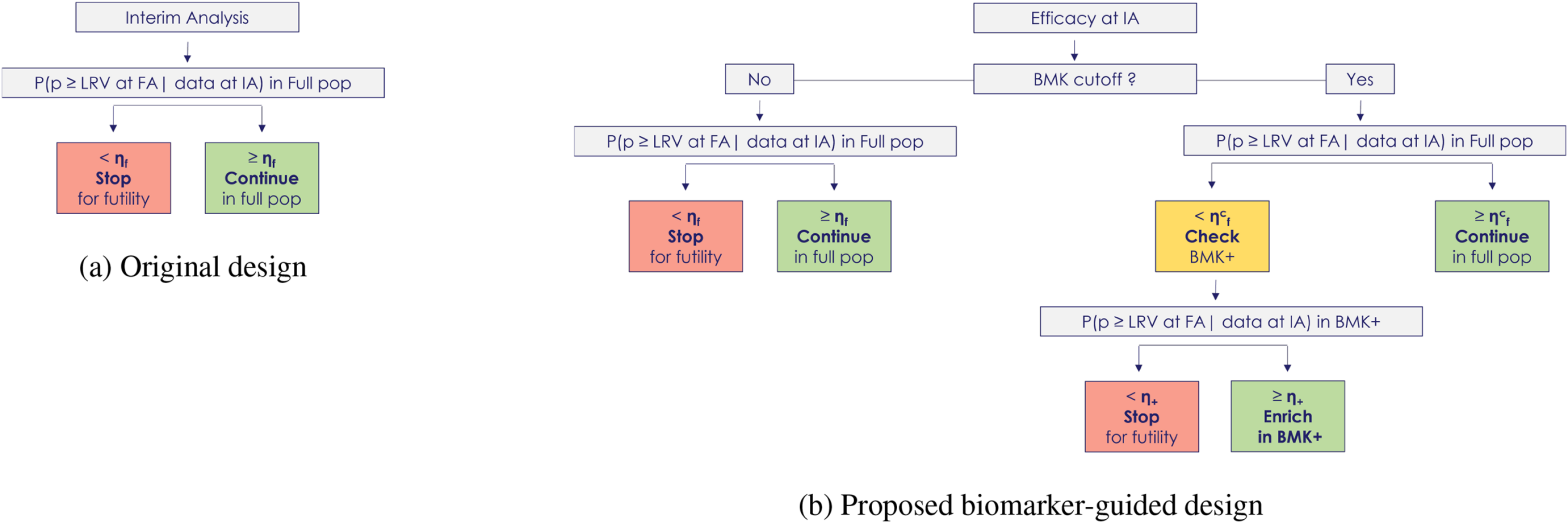
Panel (a): schematic of the decision rules at the IA for the original design. Panel (b): schematic of the decision rules at the IA for the biomarker‐guided design.

At the interim analysis, the decision rules are based on the predictive probability of meeting the success criteria [12] at the final analysis based on the data from the full population observed until that point. These are represented in Figure 1a.

If there is enough evidence to reach the success criteria at the end of the study based on the data observed at the IA, thus ensuring that the probability of reaching success at the end of the trial ($Pr_{Go}$) is larger than a pre‐specified threshold $\eta_{f}$, that is

(1) $$Pr_{Go}=\overset{N_{f}-n_{f}}{\underset{r_{N_{f}-n_{f}}=0}{\sum }}((1-P(p<LRV\;|\;D_{n_{f}})>\alpha_{LRV})\times \varphi_{f})\geq \eta_{f}$$

with $p\;|\;D_{n_{f}}∼Beta(0.5+r_{n_{f}}+r_{N_{f}-n_{f}},0.5+N_{f}-r_{n_{f}}-r_{N_{f}-n_{f}})$ and φf=Nf−nfrNf−nf×Beta(rnf+rNf−nf,Nf−rnf−rNf−nf)Beta(.5+rnf,.5+nf−rnf), where $Beta(a,b)=\int_{0}^{1}t^{\left(a-1\right)}{\left(1-t\right)}^{\left(b-1\right)}dt$ is a beta‐function, then the study continues to the second stage where additional patients are recruited from the full population, otherwise, the study stops.

## 4 Proposed Biomarker‐Guided Design

In order to introduce the proposed design, some additional notations are needed. Let $n_{+}$ denote the number of recruited patients which are biomarker‐positive (the subscript + refers to the sub‐population of BMK+ patients) at the interim analysis. Let $N_{+}$ be the total number of patients at the final analysis when at the interim analysis the recruitment is restricted to the sub‐population. The prevalence of patients who are BMK+ is denoted by $q_{+}$.

For the proposed design, the success criteria at the final analysis are the same as per the original design of the motivating trial. These are computed as described in Section 3.2, where $i=N_{+}$ in the case the trial proceeds to the final stage in the restricted sub‐population.

At the IA, instead, the decision rules differ depending on whether a biomarker cutoff value can be found or not. Thus, the first step of the study is to determine whether a cutoff of the biomarker, which divides the population into two groups (those who respond better and those who respond less to the drug), can be declared at the interim analysis.

### 4.1 Biomarker Cutoff Identification

We assume to not have much prior information on the biomarker‐response shape of the curve and we aim to avoid the issue of misspecifying the correct model. Thus, we propose to declare the presence of ‘cutoff’ or ‘no cutoff’ by fitting a step function and by checking if there is at least a certain percentage (pp%) of the population above the cutoff value and the difference between biomarker positive and biomarker negative responses are at least ${\text{diff}}_{thr}\%$ in response rate between the two groups. Indeed, the difference in response rates between the two groups should be large enough to be interesting to explore. The choice of this parameter ${\text{diff}}_{thr}\%$ can be discussed with the clinical team and it can be specific to the trial. Simulations to understand how different choices could affect the operating characteristics of the trial might be needed. More details on this are provided in Section 5.2. Then, if the probability of this is higher than $p_{thr}$, that is

$$P[P(p_{1}>p_{0})>{\text{diff}}_{thr}]>p_{thr},$$

where $p_{1},p_{0}$ being the response rates in the biomarker‐positive and negative patients, respectively, then we declare the presence of a cutoff. The response rates $p_{1},p_{0}$ are assumed to be drawn from a beta distribution with improper prior, that is a beta with prior parameters equal to zero. The step function is fitted following the idea proposed by Vradi et al. [13] but with the Ordinary Least Squares method, which minimizes the sum of squares, instead of a Bayesian model method due to convergence issues with the Bayesian model with a small sample size. A Bayesian approach to modelling the biomarker cutoff may be preferred if prior data are informing this. If convergence issues exist, they could be resolved in other ways. For example, using weakly informative, but reasonable, priors for the location of the biomarker cutoff.

### 4.2 Decisions at the Interim Analysis

At the IA, the decision rules are based on the predictive probability of meeting the success criteria [12] at the final analysis based on the data observed until that point. A schematic of the decision rules at the IA for the proposed design is summarised in Figure 1b.

If the presence of a cutoff can not be declared, then the decision rules are the same as per the original design described in Section 3.2. Thus, if there is enough evidence to reach the success criteria at the end of the study based on data from the full population observed at the IA, that is ensuring that the probability of reaching success at the end of the trial is larger than a pre‐specified threshold $\eta_{f}$, as described in Equation (1), then the study continues to the second stage where additional patients are recruited from the full population, otherwise the study stops.

If a cutoff value can be declared and if there is enough evidence to reach the success criteria at the end of study considering the full population based on the data observed at the IA, thus ensuring that the probability of reaching success at the end of the trial is larger than a pre‐specified threshold $\eta_{f}^{c}$, that is $Pr_{Go}\geq \eta_{f}^{c}$, where $Pr_{Go}$ is computed as described in Equation (1), then the study proceeds to the next stage where additional patients are recruited from the full population. If instead the success in the full population cannot be predicted with sufficient confidence, we look at the predicted success in the biomarker‐defined subgroup. Thus, if there is evidence of success at the FA on the sub‐population, in other words the probability of reaching success in the sub‐population at the end of the trial is larger than a pre‐specified threshold $\eta_{+}$ that is

$$\overset{N_{+}-n_{+}}{\underset{r_{N_{+}-n_{+}}=0}{\sum }}((1-P(p<LRV\;|\;D_{n_{+}})>\alpha_{LRV})\times \varphi_{+})\geq \eta_{+},$$

with $p\;|\;D_{n_{+}}∼Beta(0.5+r_{n_{+}}+r_{N_{+}-n_{+}},0.5+N_{+}-r_{n_{+}}-r_{N_{+}-n_{+}})$ and φ+=N+−n+rN+−n+×Beta(0.5+rn++rN+−n+,0.5+N+−rn+−rN+−n+)Beta(.5+rn+,.5+n+−rn+), and $Pr_{Go}<\eta_{f}^{c}$, where $Pr_{Go}$ is computed as per Equation (1), then the development continues and recruitment is restricted to the sub‐population of BMK+ patients. If those conditions are not satisfied, then the study is stopped.

## 5 Simulation Study

In this section, we evaluate the performance of the proposed approach using a step function to describe the response‐biomarker relationship and compare it with the competing approach described in Section 4.2. The numerical results are found using the software R [14] (version 4.3.0, running under Windows 10 x64) and 5000 simulation replicates.

### 5.1 Setting

We consider the clinical trial setting described in Section 2. The interim analysis is performed after observing 14 patients and a total of 27 patients might be recruited by the end of the study. The distribution of the biomarker was determined combining all indications included in Servier's trial. This is supposed to be normally distributed and centered around 3.46 with a known standard deviation, that is $X∼N(\mu_{X}=3.46,\sigma_{X}=1.3)$. Data are generated following a step function relationship between the patient's outcome Y and the biomarker X, that is:

$$p=\left\{\begin{array}{l} p_{1}\;\text{if}\;X>c\\ p_{0}\;\text{if}\;X\leq c \end{array}\right.\;Y∼Bin(p_{k}),k\in \{0,1\}$$

where $p_{1},p_{0}$ are the response rates above and below the true cutoff c, which is specified as a quantile of X in regards of a pre‐specified prevalence of patients which are BMK+ ($q_{+}$).

The considered scenarios, summarised in Table 1, were chosen in order to explore the impact of the prevalence $q_{+}$, the impact of the difference between biomarker‐positive and negative response rates, the impact of considering different TV and LRV values at the final analysis and the control of false positive under the null scenarios, that is when there is no difference in response rates for biomarker‐positive and biomarker‐negative patients. The scenarios will be labeled as: NPHR non‐predictive with high response rate scenario; NPMR non‐predictive with medium response rate scenario; NPLR non‐predictive with low response rate scenario; PHR predictive with high response rate scenario; PMR predictive with medium response rate scenario. The subscripts $q_{1}$ and $q_{2}$ differentiate the scenarios considering different prevalences of BMK+ patients. The TV and LRV values are set to 15% and 5%, respectively. Sensitivity analyses with different values of TV and LRV are presented in Section 5.6.

**TABLE 1:** True response rates in the BMK‐ subgroup ($p_{0}$) and in the BMK+ subgroup ($p_{1}$). The prevalence of BMK‐positive patients is indicated by $q_{+}$ and the overall response rate in the full population is p.

Scenario	$p_{0}$	$p_{1}$	Prevalence $q_{+}$	Overall p
${\text{NPHR}}_{q_{1}}$	15%	15%	50%	15%
${\text{NPHR}}_{q_{2}}$	15%	15%	30%	15%
${\text{NPMR}}_{q_{1}}$	5%	5%	50%	5%
${\text{NPMR}}_{q_{2}}$	5%	5%	30%	5%
${\text{NPLR}}_{q_{1}}$	0%	0%	50%	0%
${\text{NPLR}}_{q_{2}}$	0%	0%	30%	0%
${\text{PHR}}_{q_{1}}$	5%	25%	50%	15%
${\text{PHR}}_{q_{2}}$	2.5%	27.5%	30%	10%
${\text{PMR}}_{q_{1}}$	5%	15%	50%	10%

The scenarios are constructed considering different response rates $p_{0}$ and $p_{1}$ and different values of the prevalence of BMK+ patients (50% or 30% prevalence) to obtain an overall response rate between the TV and LRV values. For example, the ${\text{PHR}}_{q_{1}}$ scenario considers $p_{0}=5\%$, $p_{1}=25\%$ and $q_{+}=50\%$. Thus the overall response rate p, is computed as $p=p_{0}\times (1-q_{+})+p_{1}\times (q_{+})=15\%$. As this work is motivated by the real clinical trial that the authors are involved with, the assumptions for the evaluation were informed by the relevant clinical knowledge available. Specifically, the prevalence of 30% was informed by the discussion with clinicians and the study from Fong et. al [15], where the first tertile was selected as optimal to differentiate patients with low expression from high expression of the biomarker. The prevalence of 50% was also investigated to explore the operating characteristics of the proposed design when a larger number of patients could benefit from the drug. Nevertheless, also a lower prevalence (15%) was explored and results are presented in Section 3.3 of the Supporting Information.

For the simulation study, the thresholds used at the final analysis are set as $\alpha_{TV}=10\%$ and $\alpha_{LRV}=80\%$ following the conventional confidence levels described in Frewer et al. [11]. Instead, the thresholds used for decision‐making at the time of the interim analysis are described in Section 5.4.

### 5.2 Comparison of Methods to Declare a Cutoff

In this section, the approach proposed in Section 4.1 for the biomarker cutoff identification will be compared with other two approaches to declare the presence of ‘cutoff’ or ‘no cutoff’:

1. ‘Naive’ approach that checks whether there is a certain number of responses in at least a certain percentage of the population, without dividing the population into two groups. This means that using this approach, we declare that there is a ‘cutoff’ if at least 15% of responses (that for the motivating trial setting is at least 3 responses in 14 patients) in at least pp% of the population;
2. ‘Naive with step function’ approach that checks whether there is a certain number of responses in at least a certain percentage of the population which has a biomarker measure above a value that is found by fitting a step function. This approach does not require that the difference in response rates between biomarker‐positive and biomarker‐negative patients is large enough. This means that using this approach, we find a cutoff by fitting a step function and declare that there is a ‘cutoff’ if there are at least 15% of responses in at least pp% of the population with this cutoff value.

In the setting of the simulation study, the three approaches will be compared under two specific scenarios. The true cutoff of the biomarker is set at its median value. Data are generated following a step function and one scenario considers the response rates above and below the true cutoff to be 15% (${\text{NPHR}}_{q_{1}}$ scenario), while the other scenario considers the response rates to be 25% and 5% above and below the cutoff respectively (${\text{PHR}}_{q_{1}}$ scenario). For the comparison, values of 10%, 20% and 30% are used for the prevalence of population above the cutoff (pp%) and, for the approach proposed in Section 4.1, values of 0.1, 0.15 and 0.2 for ${\text{diff}}_{thr}$ are used and values of 0.7, 0.8 and 0.9 are used for $p_{thr}$.

The results are summarised in Table 2, where the ‘Probability based’ approach refers to the one described in Section 4.1. The ‘Naive’ method provides the highest probability (above 80%) to find a cutoff under the alternative scenario compared to the other approaches, however, it is also the one that leads to the highest false conclusions (above 70%) of declaring the presence of a cutoff under the null scenario.

**TABLE 2:** Probability to find a cutoff for the three considered approaches. In bold the chosen gain value is highlighted. The percentage of the population above the cutoff is pp%, the threshold to compare the difference in response rate is ${\text{diff}}_{thr}$ and the probability threshold is $p_{thr}$.

Approach	pp%	${\text{diff}}_{thr}$	$p_{thr}$	False positives under ${\text{NPHR}}_{q_{1}}$	True positives under ${\text{PHR}}_{q_{1}}$	Gain: True—false positives
‘Naive’	10	—	—	0.74	0.83	0.10
	20	—	—	0.73	0.83	0.11
	30	—	—	0.74	0.82	0.09
‘Naive with step function’	10	—	—	0.41	0.65	0.23
	20	—	—	0.35	0.56	0.20
	30	—	—	0.27	0.37	0.11
‘Probability based’	10	0.1	0.7	0.29	0.55	0.26
			0.8	0.24	0.50	**0.26**
			0.9	0.14	0.37	0.23
		0.15	0.7	0.24	0.50	0.26
			0.8	0.16	0.40	0.24
			0.9	0.10	0.28	0.18
		0.2	0.7	0.19	0.42	0.22
			0.8	0.13	0.33	0.20
			0.9	0.07	0.21	0.14
	20	0.1	0.7	0.23	0.45	0.23
			0.8	0.19	0.41	0.22
			0.9	0.13	0.32	0.19
		0.15	0.7	0.19	0.42	0.24
			0.8	0.13	0.33	0.19
			0.9	0.08	0.25	0.18
		0.2	0.7	0.13	0.33	0.20
			0.8	0.10	0.27	0.17
			0.9	0.06	0.18	0.12
	30	0.1	0.7	0.14	0.31	0.17
			0.8	0.13	0.29	0.15
			0.9	0.09	0.23	0.14
		0.15	0.7	0.13	0.28	0.16
			0.8	0.10	0.24	0.14
			0.9	0.05	0.15	0.10
		0.2	0.7	0.10	0.23	0.13
			0.8	0.06	0.18	0.12
			0.9	0.04	0.12	0.08

Our aim is to maximise the probability of finding a cutoff under the alternative scenario but at the same time being able to control the number of false conclusions. Thus, the three approaches are compared in terms of ‘Gain’, that is the difference between the probability of declaring to find a cutoff under the alternative and the null scenarios. The configuration of parameters that leads to the highest gain (26%) for the setting where $p_{thr}$ is set to be equal to the threshold $\alpha_{LRV}$ = 80%, is when $pp=10\%,{\text{diff}}_{thr}=0.1$. Thus, a cutoff value is declared to be found if we are 80% certain that there is 10% difference in response rates between the two groups in at least 10% of the population. This criterion will be used in the simulation study.

### 5.3 Metrics and Operating Characteristics of the Design

The following metrics will be explored in the simulation study for proposed design:

- the decision probabilities at the interim analysis: probability to stop for futility when a cutoff can or can not be declared, the probability to continue to the second stage with the full population and the probability to continue with the sub‐population of BMK+ patients;
- probability to Go, No Go, Consider at the FA with full or sub‐population or regardless of the population conditional on continuing to the second stage;
- overall decisions at the end of the trial: probability to Go, No Go and Futility and Consider regardless of the population and the timing of the analysis;
- the probability of declaring a cutoff, the average value for the biomarker cutoff and its 25th and 75th percentiles and the expected sample size (ESS), that is the average number of patients that are recruited at the end of the trial.

For the original design, the overall decisions at the end of the trial will be explored and compared to those of the proposed design.

### 5.4 Thresholds for Decision‐Making at the Interim Analysis

In order to evaluate the operating characteristics of the proposed design under the different scenarios, the values of the thresholds used in the interim analysis for decision‐making need to be chosen. The setting of the simulation study described in Section 5.1 is considered here.

Firstly, the threshold $\eta_{f}$ was chosen for the original design of the motivating trial depending on the TV and LRV values used at the final analysis. The threshold $\eta_{f}$ is set to be equal to 0.1 under the scenarios that consider TV/LRV values of 15%/5% in order to ensure at the time of the interim analysis to proceed to the final analysis if at least 1 response (and this corresponds to 7%, that is 1 response out of 14 patients, and it is greater than LRV) is observed.

Secondly, considering the chosen value of $\eta_{f}=0.1$, several values of $\eta_{f}^{c},\eta_{+}$, were explored in order to investigate how the decision‐making would change for values of these thresholds varying from 0 to 1. The probability to Go at the FA and to enrich in the BMK+ population at the IA for different values of $\eta_{f}^{c}$ and $\eta_{+}$ under the ${\text{PHR}}_{q_{1}}$, ${\text{NPHR}}_{q_{1}}$ and ${\text{NPMR}}_{q_{1}}$ scenarios are represented in the Figure provided in Section 2 of the Supporting Information. The final values of $\eta_{f}^{c}=0.9$ and $\eta_{+}=0.75$ were chosen in order to ensure an overall probability to Go above 75% under the ${\text{PHR}}_{q_{1}}$ and ${\text{NPHR}}_{q_{1}}$ scenarios and an overall probability to Go below 15% under the ${\text{NPMR}}_{q_{1}}$ scenario, and maximising the probability to enrich under ${\text{PHR}}_{q_{1}}$, while minimizing the enrichment under ${\text{NPHR}}_{q_{1}}$ and ${\text{NPMR}}_{q_{1}}$ scenarios. Thus, these pre‐specified thresholds will be used in the simulation study presented in Section 5.5.

### 5.5 Numerical Results of the Simulation Study

**FIGURE 2:**
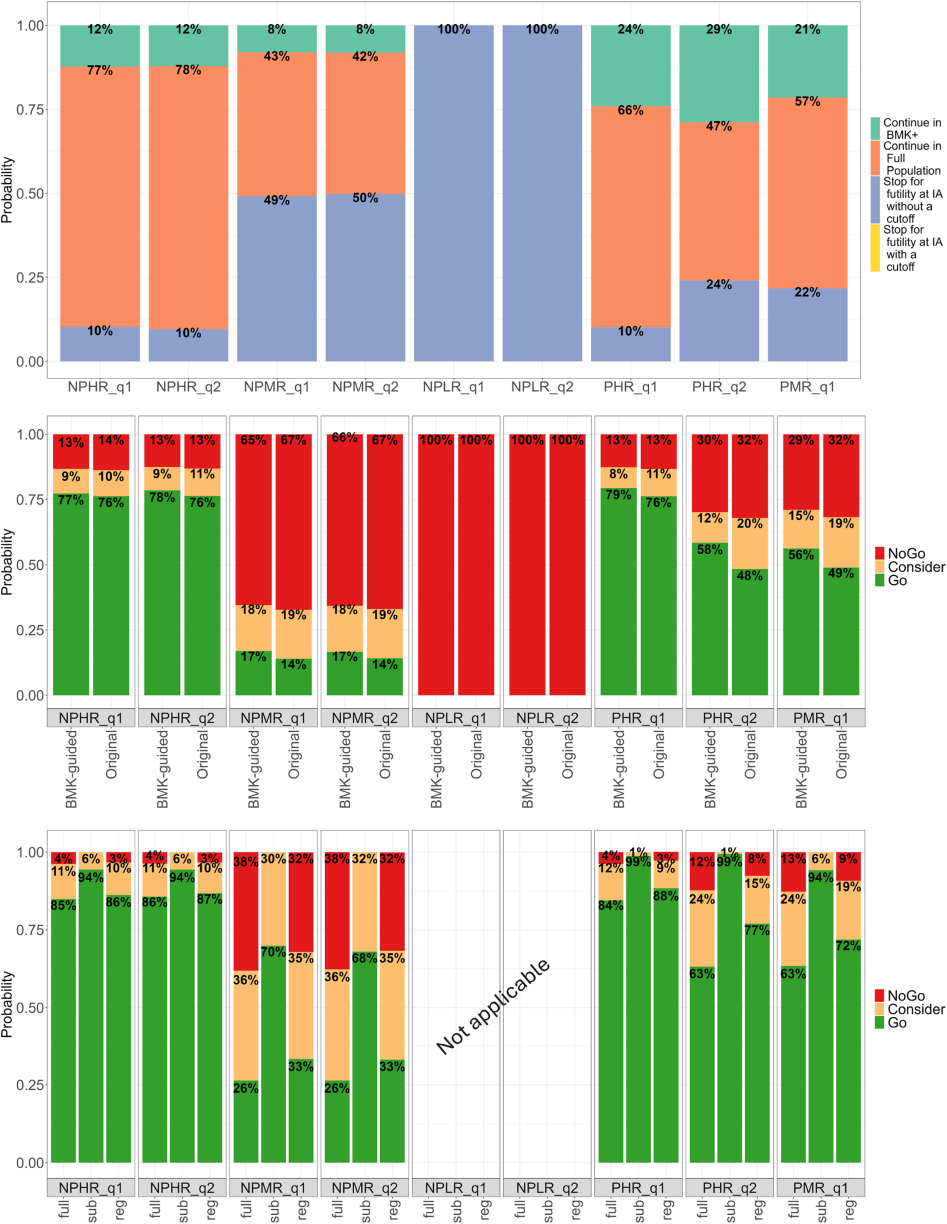
Top Panel: decisions at the IA for the proposed design. Middle Panel: overall decisions at the end of the trial for both designs regardless of the population and the timing of the analysis for all scenarios. Bottom Panel: conditional decisions at the end of the trial for the proposed design with full or sub‐population or regardless of the population conditional on continuing to the second stage for all scenarios.

Figure 2 provides the results for the decision‐making for all scenarios described in Table 1. In the top panel, the results of the decision‐making at the interim analysis for the proposed design are provided. The middle panel provides the overall decisions at the end of the trial regardless of the population and the timing of the interim analysis for both designs, while the bottom panel shows the probabilities at the FA conditional on continuing to the second stage for the proposed design.

Finally, Table 3 provides the probability of declaring a cutoff, the ESS and the average value of the cutoff for all considered scenarios.

**TABLE 3:** Probability to find a cutoff, expected sample sizes (ESS) with its 25th and 75th percentiles and average value for the biomarker cutoff with its 25th and 75th percentiles for all scenarios.

Scenario	Probability of declaring a cutoff	ESS (25th, 75th percentiles)	Average estimated cutoff (25th, 75th percentiles)
${\text{NPHR}}_{q_{1}}$	0.25	23 (17, 27)	3.45 (2.73, 4.15)
${\text{NPHR}}_{q_{2}}$	0.24	22 (17, 27)	3.48 (2.76, 4.21)
${\text{NPMR}}_{q_{1}}$	0.09	18 (15, 20)	3.44 (2.94, 3.94)
${\text{NPMR}}_{q_{2}}$	0.09	18 (15, 20)	3.46 (2.98, 3.95)
${\text{NPLR}}_{q_{1}}$	0.00	—	—
${\text{NPLR}}_{q_{2}}$	0.00	—	—
${\text{PHR}}_{q_{1}}$	0.49	22 (17, 27)	3.91 (3.42, 4.49)
${\text{PHR}}_{q_{2}}$	0.42	20 (15, 27)	4.11 (3.55, 4.66)
${\text{PMR}}_{q_{1}}$	0.31	20 (16, 27)	3.77 (3.21, 4.35)

Firstly, we describe the results under the null scenarios, which are the scenarios where there is no difference in response rates between the BMK+ and BMK‐ patients.

At the interim analysis, under the ${\text{NPHR}}_{q_{1}}$ scenario, there is 10% chance to stop for futility when a cutoff value can not be declared, 77% probability to continue to the second stage with the full population and 12% probability to enrich in the BMK‐positive sub‐population. Similar probabilities are also observed under ${\text{NPHR}}_{q_{2}}$. When the overall response rate is lower (${\text{NPMR}}_{q_{1}}$ scenario), then there is a higher chance to stop for futility (49%) and a lower chance to continue with the full population (43%). The probability to enrich in this case is 8%. Similar probabilities are also observed under ${\text{NPMR}}_{q_{2}}$. In the ${\text{NPLR}}_{q_{1}}$ and ${\text{NPLR}}_{q_{2}}$ scenarios, where the overall response is 0%, the proposed design always stops at the IA for futility.

Regarding the overall decision probabilities at the end of the trial, the proposed biomarker‐guided design and the original design present similar probabilities to Go (difference of 1%, 3% under scenarios ${\text{NPHR}}_{q_{1}}$, ${\text{NPMR}}_{q_{1}}$ respectively), to Consider (difference of 1% under scenarios ${\text{NPHR}}_{q_{1}}$, ${\text{NPMR}}_{q_{1}}$ respectively) and to No Go (difference of 1%, 2% under scenarios ${\text{NPHR}}_{q_{1}}$, ${\text{NPMR}}_{q_{1}}$ respectively). For the proposed design, under scenarios ${\text{NPHR}}_{q_{1}}$, the probability to Go is around 77% while it decreases when the overall response rate decreases. Under ${\text{NPMR}}_{q_{1}}$ the probability to Go is around 17%. Additional information on the probability to Go, No Go and Consider at the FA with full or sub‐population or regardless of the population conditional on continuing to the second stage, are provided in the bottom panel of Figure 2. The probability to Go at the FA with the sub‐population, conditional on going to the second stage, is always higher than that with the full population. This is because when we identify a sub‐population, we have already high confidence that the sub‐population responds better (has higher response rate) than the full population. Even though, under the null scenarios, identifying a population that responds better is a false conclusion. Similar overall patterns are observed for the null scenarios with a different prevalence, that are ${\text{NPHR}}_{q_{2}}$, ${\text{NPMR}}_{q_{2}}$.

Regarding the probability of declaring a cutoff value, Table 3 shows that there is a cutoff in 25% or 9% under scenarios ${\text{NPHR}}_{q_{1}}$, ${\text{NPMR}}_{q_{1}}$, respectively. In all these scenarios, the average value of the cutoff is around 3.44 and 3.45, thus very close to the true expected value of the biomarker X, that is $\mu_{X}=3.46$.

Regarding the scenarios where there is a difference in response rates between biomarker‐positive and negative patients, in scenarios ${\text{PHR}}_{q_{1}}$ and ${\text{PMR}}_{q_{1}}$, there is 10% and 22% chance to stop for futility at the interim analysis respectively, and the probability to enrich, and thus, continue in the sub‐population is 24% and 21%, respectively. In terms of final decisions at the end of the trial, when the response rate is higher (scenario ${\text{PHR}}_{q_{1}}$), there is a difference of around 3% in terms of probability to Go between the proposed design and the classical approach (from 79% to 76% respectively), while the two designs present the same probability of not proceeding to the next phase of drug development (13% of No Go). When the response rate is lower (scenario ${\text{PMR}}_{q_{1}}$), the two designs present both a lower probability to Go (56% and 49%, for the biomarker‐guided and the classical approach respectively) and a higher probability to stop the drug development (around 29% and 32%, for the biomarker‐guided and the classical approach respectively). Under both of these scenarios, there is gain in using the proposed design compared to the classical one, as at the end of the trial there is a similar chance to stop the development but a higher probability to continue. Indeed, the bottom panel of Figure 2 shows that under the ${\text{PHR}}_{q_{1}}$ and ${\text{PMR}}_{q_{1}}$ scenarios, there is a high chance to Go at the FA with the sub‐population (99% and 94% respectively) conditional to the fact that the trial continues to the second stage (probability to enrich is 24% and 21%, respectively, for these two scenarios).

When the prevalence of BMK‐positive patients is decreased by 20% (scenario ${\text{PHR}}_{q_{2}}$), then there is a higher gain in terms of probability to Go (a difference of around 10% between the biomarker‐guided and the classical approach) compared to the scenario ${\text{PHR}}_{q_{1}}$ where the prevalence of biomarker was 50%. In ${\text{PHR}}_{q_{2}}$ scenario, the probability to stop the development is around 30% and 32% for biomarker‐guided and the classical approach and there is a 29% chance to continue to enrich at the interim analysis.

In terms of probability of declaring the presence of a cutoff, it increases when the response rates in the BMK‐positive subgroup increases (31% or 49% under ${\text{PMR}}_{q_{1}}$ or ${\text{PHR}}_{q_{1}}$ respectively). When the prevalence of BMK‐positive patients is 30% (scenario ${\text{PHR}}_{q_{2}}$), then the probability of declaring a cutoff is slightly smaller (42%). In terms of the mean estimate of the cutoff value, the estimations are not too far off the true values (3.91 and 3.77 when the true value is 3.46 under ${\mathrm{PMR}}_{q1}$ and ${\mathrm{PHR}}_{q1}$ scenarios, respectively, and 4.11 when the true value is 4.14 under ${\mathrm{PHR}}_{q2}$ scenario).

### 5.6 Sensitivity Analyses

Sensitivity analyses are explored for the same response rates as described in Table 1, but with a prevalence ($q_{+}$) of 15% or considering an interim analysis done after a slightly larger number of patients (18 instead of 14) have been treated. These are presented in Sections 3.3 and 3.4 of the Supporting Information. Similar patterns to those described in Section 5.5 can be observed here. Overall, considering a prevalence of 15%, at the end of the trial, a gain in the probability to Go up to 14% can be observed for the BMK‐guided design compared to the classical approach, while in terms of the probability of stopping (No Go) at the end of the trial, the classical approach presents always higher probabilities (up to 4%) compared to the biomarker‐guided approach for all considered scenarios. Regarding the results with the interim analysis done after treating a larger number of patients, we can observe that overall the probability to Go is higher at the end of the trial for all considered scenarios, compared to the case where only 14 patients are considered at the time of the interim analysis, and with a gain up to 11% for the BMK‐guided design compared to the classical approach. Overall, only under some scenarios there is a small probability (up to 3%) to Consider at the end of the trial. Under the null scenarios, it can be observed that overall the probability to enrich is similar or slightly smaller compared to the case where only 14 patients are considered at the time of the interim analysis.

Further sensitivity analyses are run considering different values of prevalence ranging from 0.2 to 0.8, different values of response rates in BMK‐positive patients ($p_{1}$) ranging from 0.2 to 0.7 when the BMK‐negative subgroup has no or half of the effect of the BMK‐positive subgroup ($p_{0}=0$ or $p_{0}=0.5\times p_{1}$, respectively). These are summarised in Section 3 of the Supporting Information. When there is no effect in the BMK‐negative subgroup (see Figure 3 in Section 3 of the Supporting Information), up to 19% gain in the probability to Go can be reached when the prevalence of biomarker‐positive patients is low (20%) but the response rate is 30%. When instead the response rate in the biomarker‐negative patients is half of the biomarker‐positive, the gain is up to 5% when the prevalence is between 30% and 50% and the response rate in the biomarker‐positive patients is 20%. In terms of probabilities to consider at the end of the trial, we do observe some slightly differences (around 13%) when there is no effect in the biomarker‐negative subgroup and the response rate of the biomarker‐positive patients is low (30%). In terms of the probability of stopping (No Go) at the end of the trial, the classical approach presents always higher probabilities (up to 10%) compared to the biomarker‐guided approach for all considered scenarios.

In addition, the same analyses have been carried out considering TV = 30% and LRV = 19%. In this setting, when there is no effect in the BMK‐negative subgroup (see Figure 4 in Section 3 of the Supporting Information), up to 60% gain in the probability to Go can be reached when the prevalence of biomarker‐positive patients is low (20%) but the response rate is high (80%). When instead the response rate in the biomarker‐negative patients is half of the biomarker‐positive, the gain is up to 15% when the prevalence is between 20% and 30% and the response rate in the biomarker‐positive patients is 30% or 40%. In terms of probabilities to consider at the end of the trial, we do observe some slightly differences (around 10%) when there is no effect in the biomarker‐negative subgroup and the response rate of the biomarker‐positive patients is low (20%). In terms of the probability of stopping (No Go) at the end of the trial, the classical approach presents always higher probabilities (up to 51%) compared to the biomarker‐guided approach for all considered scenarios.

In addition to those analyses, further five additional scenarios have been investigated: the NPMR_2 and NPLR_2 scenarios that are the null scenarios when the TV and LRV values are set to 30% and 19% respectively; the ${\text{PHR\_2}}_{q_{1}}$ and ${\text{PHR\_2}}_{q_{2}}$ which have higher response rates than ${\text{PHR}}_{q_{1}}$ and ${\text{PHR}}_{q_{2}}$ scenarios and the TV and LRV values are set to 30% and 19%; the ${\text{SPHR}}_{q_{2}}$ which is the same scenario in terms of response rates and prevalence as ${\text{PHR}}_{q_{2}}$ but it considers 20 patients at the time of the interim analysis instead of 14. These are summarised in Section 3.1 of the Supporting Information. It was observed that higher TV/LRV does not impact significantly the operating characteristics. As expected, the scenario with a very marked difference between BMK+ and BMK‐ (50% difference) shows very good operating characteristics with almost 80% to declare a cutoff, even with only 14 patients at IA. In addition, if more patients are considered at IA (20 vs. 14), then there is an increase in the probability of detecting a signal (+15%). Some further simulations could be done to define an optimal time for the interim analysis for this type of design.

Finally, sensitivity analyses have been conducted around the distribution of the biomarker. In particular, two gamma distributions have been considered: the so called ‘Less Skewed’, which has the same mean $\mu_{X}=3.46$ as the normal distribution considered in the main results of Section 5 and the so called ‘Skewed’, which has the same variance $\sigma_{x}^{2}=1.69$ as the normal distribution considered in the main results of Section 5. These are represented in Figure 5 in Section 3.2 of the Supporting Information. The same scenarios as described in Table 1 in Section 5 are considered. The results are summarised in Section 3.2 of the Supporting Information, but very similar patterns and conclusions to the normal biomarker case can be drawn here between scenarios and designs.

## 6 Discussion

At the PoC stage, a key question is whether to recruit patients into the clinical study based on a biomarker that might predict treatment response, particularly when the biomarker is continuous. There are three options to consider. The first one consists on considering all patients, without any restriction on the population. With this option, there is a risk to miss a signal if the treatment benefit is limited to a biomarker subgroup. A second option is to consider biomarker‐positive patients based on an arbitrary cutoff. Nevertheless, there is still a risk of missing a signal if the biomarker cutoff is not well established. Thirdly, an adaptive BMK‐guided design could be considered. Here there is a risk to make a wrong interim decision due to a limited sample size at the IA.

Before implementing a BMK‐based design, it is critical to assess the potential prognostic value of the biomarker of interest. In oncology PoC studies, which often rely on single‐arm designs, such evaluations are essential. In this work, an early‐phase biomarker‐guided design has been proposed and this can be used when we have a strong rationale for the predictiveness of the biomarker but not a strong prior on the biomarker cutoff, as is often the case in early development stages (relying primarily on preclinical data). If an efficacy signal is identified in a biomarker subgroup at the PoC stage, then the next clinical trial could be designed to refine the biomarker cutoff on a larger sample size. Moreover, the proposed design is particularly beneficial when we expect a marked differential benefit according to the biomarker value and when the prevalence of biomarker‐positive is low.

The proposed adaptive design has been shown to outperform the non‐adaptive approach with a gain up to 60% in the overall probability to Go compared to the classical design when there is a true biomarker cutoff. It has been observed that there is a limited false enrichment when there is no true biomarker cutoff, and overall, the probability to Go/No Go is almost the same as per the classical design. In addition, if a cutoff could be declared to be found, then, in most of the considered scenarios, we do not stop for futility.

We have investigated the operating characteristics of the proposed biomarker‐guided design in the setting of a motivating trial. The motivating study is still under consideration and has not yet commenced. It is also our plan to publish the details of the biomarker‐related analysis when this study comes to completion. Sensitivity analyses have been performed considering different variations of the trial setting and different distributions of the biomarker. The proposed design has shown to provide consistent results across the considered settings. Despite this, further evaluations might be necessary in other clinical trial settings. For example, the specific choices of the thresholds for decision‐making at the interim analysis might vary or need to be re‐evaluated in other clinical trial settings. In this work, the proposed thresholds at the interim analysis can also be converted into the number of observed responses. Figure 1 in Section 1 of the Supporting Information describes the decision rules at the IA for the proposed design for the setting of the motivating trial in terms of the observed number of responses. Additionally, the timing of the interim analysis could be further investigated. Some sensitivity analyses have been conducted considering a larger number of patients at the interim analysis (18 patients instead of 14). Similar patterns and overall conclusions can be drawn here regarding the advantages of considering a biomarker‐guided design compared to the classical approach.

In this work, we have proposed a method in order to declare the presence or not of a biomarker cutoff at the time of the interim analysis. We have compared it with two other approaches. The proposed approach seemed to perform quite well under all analysed scenarios and considering such a small sample size. However, further work is needed to compare this approach to other classical approaches (e.g., Youden index [16], SIDES approach [17]).

In addition, further work is needed to compare the proposed biomarker‐guided design to other alternative designs, such as an adaptive design that excludes a subgroup of patients who do not appear to respond to treatment midway through an ongoing trial. Other additional areas of work to consider could include the assessment of the potential for continuous monitoring after an initial proportion of patients have completed the treatment and the case when there are multiple possible BMK candidates that are measured simultaneously, and one could be chosen to stratify the population.

In conclusion, the proposed design offers a robust framework for early‐phase trials, particularly in settings where biomarker predictiveness is expected but biomarker cutoff information is limited. We would like to emphasize that the primary objective of the proposed design is not to identify the biomarker‐positive group earlier as, given the very small sample size, this can be a premature decision which could restrict the subsequent study only to a subpopulation. Instead, the main objective of the proposed design is to consider restricting the rest of the study to a subpopulation only if there is (i) high confidence in the futility of the treatment in the whole population, and (ii) enough evidence of activity in the restricted subgroup. This construction allows to avoid terminating the trial of an experimental treatment that can be potentially beneficial in a smaller group of patients while still prioritizing collecting the evidence in the whole population (if it is not futile to do so). Having said that, it might be valuable to understand how early, in principle, one can accumulate enough evidence to declare a BMK‐positive population. The percentage of simulations where there was not enough information at the IA to declare a BMK‐positive, but there was at the FA is summarised in Section 3.5 of the Supporting Information. It can be observed that, for the considered scenarios in this simulation study, this probability can take values up to 6% (or up to 38% conditional on continuing in the full population at the IA). While promising, continued exploration of its adaptability and comparison with other methodologies is essential to ensure its broad applicability and effectiveness in future trials.

## Author Contributions

All authors have directly participated in the planning and execution of the presented work.

## Disclosure

The authors have nothing to report.

## Conflicts of Interest

J.G. and S.G. are the employees of Institut de Recherches Internationales Servier. G.S.‐H. is President of Saryga SAS. P.M. and A.S. served as statistical consultants for Institut de Recherches Internationales Servier and Saryga SAS.

## Supporting information




**Data S1.** Supporting Information.

## Data Availability

Data sharing is not applicable to this article as no new data were created or analyzed in this study.
