# Supplementary material for: Adaptive Biomarker‐Based Design for Early Phase Clinical Trials
Source: Stat Med. 2025 Oct 9;44(23-24):e70275. doi: 10.1002/sim.70275 (PMC12510287; doi:10.1002/sim.70275)
Supplement: Supplementary file 1 — Data S1. Supporting Information. [file SIM-44-0-s001.pdf]

Received: Added at production | Revised: Added at production | Accepted: Added at production
DOI: xxx/xxxx

ARTICLE TYPE

Supplementary material of 'Adaptive Biomarker-Based Design for Early Phase Clinical Trials'

Alessandra Serra*[1] | **Sandrine Guilleminot[3]** | **Gaëlle Saint-Hilary[2]** | **Julia Geronimi[3]** | **Pavel Mozgunov[1]**

[1]University of Cambridge, MRC Biostatistics Unit, Cambridge, United Kingdom

[2]Department of Statistical Methodology, Saryga, France

[3]Translational Statistics Department, Institut de Recherches Internationales Servier, Gif-sur-Yvette, France

Correspondence
*Corresponding author name, This is sample corresponding address.
Email: alessandra.serra@mrc-bsu.cam.ac.uk

Present address
This is sample for present address text this is sample for present address text

Identifying and quantifying predictive biomarkers is a critical issue of Precision Medicine approaches and patient-centric clinical development strategies. Early phase adaptive designs can improve trial efficiency by allowing for adaptions during the course of the trial. In this work, we are interested in adaptations based on interim analysis permitting a refinement of the existing study population according to their predictive biomarkers. At an early stage, the goal is not to precisely define the target population, but to not miss an efficacy signal that might be limited to a biomarker subgroup. In this work, we propose a one-arm two-stage early phase biomarker-guided design in the setting of an oncology trial where at the time of the interim analysis, several decisions can be made regarding stopping the entire trial early or continuing to recruit patients from the full or a selected patient population. Via simulations, we show that, although the sample size is limited, the proposed design leads to better decision-making compared to a classical design that does not consider an enrichment expansion.

KEYWORDS
Predictive; Continuous biomarker; Early Phase; Personalized medicine

1 | SCHEMATIC OF THE DESIGN

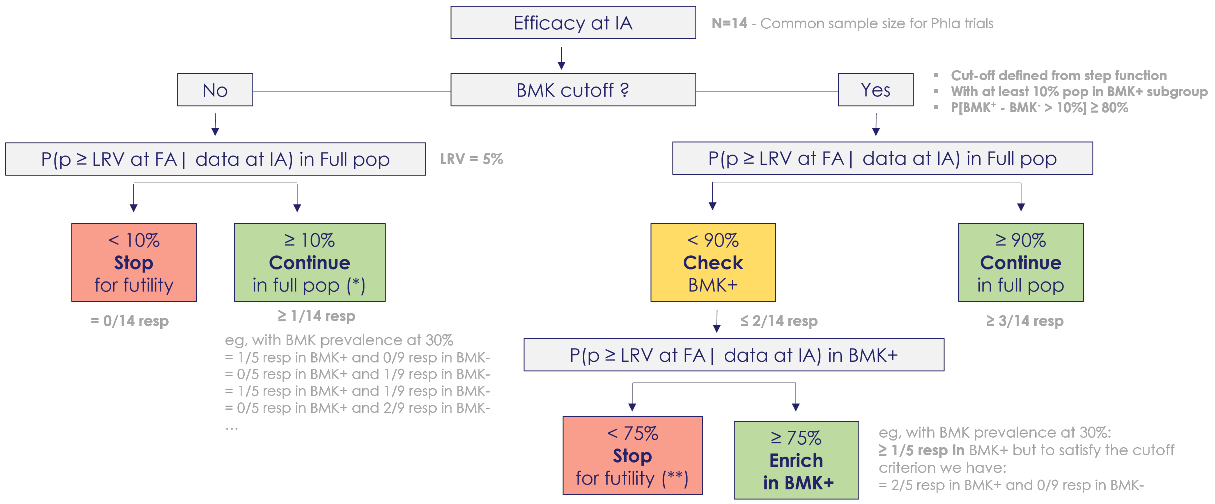

FIGURE 1 Schematic of the decision rules in terms of number of responses at the IA for the proposed design

Abbreviations: BMK, biomarker; PoC, proof of concept; IA, interim analysis; FA, final analysis; TV, target value; LRV, lower reference value

 © 2023 Copyright Holder Name

2 | CHOICE OF THE THRESHOLDS AT THE IA

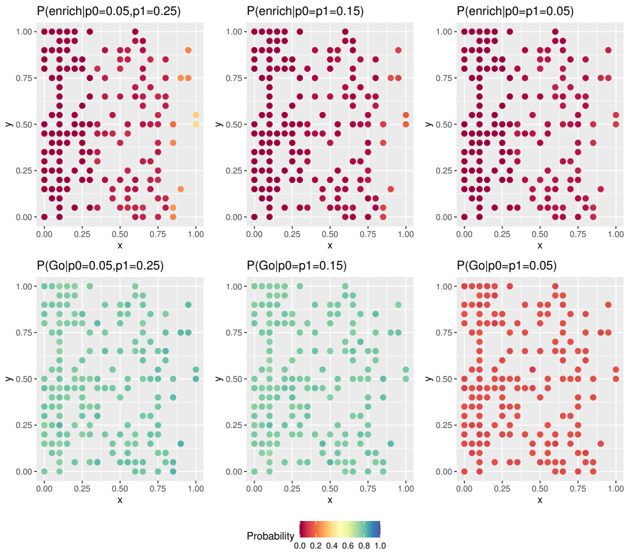

FIGURE 2 Probability to Go and to enrich for different values of η_f^c (x-axis) and η_+ (y-axis) for different values of response rates above for BMK-positive and negative patients when the overall response rate is 15% and when $\eta_f = 0.1$.

3 | SENSITIVITY ANALYSES

For the setting where TV = 30% and LRV = 19%, the threshold η_f was chosen for the original design of the motivating trial to be equal to 0.12 in order to ensure at the time of the interim analysis to proceed to the final analysis if at least 3 responses are observed (and this corresponds to 21%, that is 3 responses out of 14 patients, and it is greater than LRV). The other thresholds were not re-calibrated under this setting of TV/LRV. The calibration can be done, but as it is a sensitivity analysis we explore the operating characteristics for the same comparable parameters.

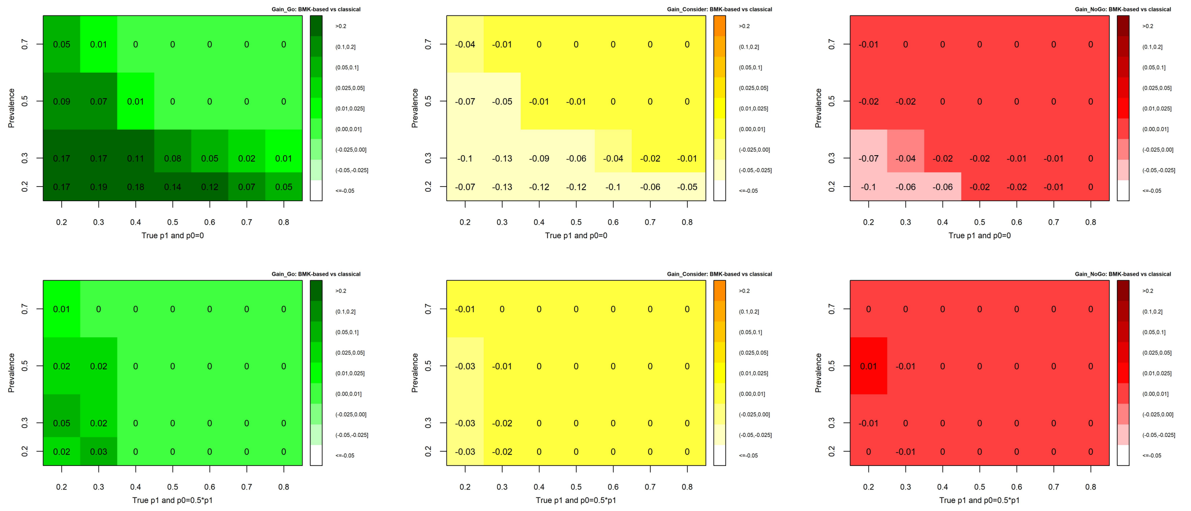

FIGURE 3 Difference in overall probabilities at the FA between BMK-design and classical design for various response rates in BMK-positive and prevalence when the response rate in BMK-negative is 0 (top row) or 50% of the rate for BMK-positive (bottom row). TV and LRV are 15% and 5% respectively.

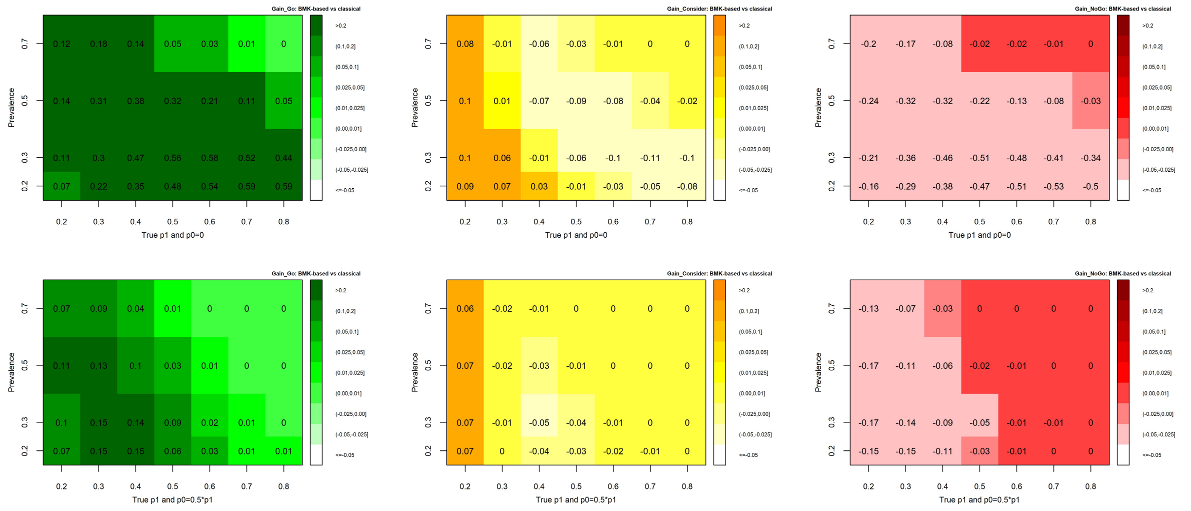

FIGURE 4 Difference in overall probabilities at the FA between BMK-design and classical design for various response rates in BMK-positive and prevalence when the response rate in BMK-negative is 0 (top row) or 50% of the rate for BMK-positive (bottom row). TV and LRV are 30% and 19% respectively.

3.1 | Additional scenarios

When the overall probability is 19% (scenario NPMR_2), there is 7% to stop for futility when a cutoff can be declared, while there is 40% chance to stop for futility when a cutoff can not be declared. In this case, there is 37% chance to continue with the full population, while 17% to enrich. In addition, in terms of overall probabilities at the end of the trial, the biomarker-guided design has 24% and 60 % probability to Go and No Go respectively, while the classical design 21% and 66% to Go and No Go respectively. Under the NPLR_2 scenario, where the overall response is 0%, we observe the same operating characteristics as for the NPLR$_{q_1}$ scenario. The proposed design always stops at the IA for futility.

It can be observed that the overall decision at the end of the trial of the proposed design under scenario PHR_2$_{q_1}$ are quite similar to the operating characteristics of the design under scenario PHR$_{q_1}$. However, under scenario PHR$_{q_1}$ there is less difference in terms of probabilities at the final analysis between the proposed design and the classical approach, while in scenario PHR_2$_{q_1}$ there is a gain of around 8% in terms of probability to go at the end of the trial compared to the classical design and a lower chance to stop the development of the drug (16% vs 21%) compare to the classical design.

Under scenario PHR_2$_{q_2}$, it can be observed that there is a higher chance to continue in the subpopulation at the interim analysis (51%) compared to all the other scenarios. Under this scenario, we can observe a higher difference in terms of probabilities at the final analysis between the proposed design and the classical approach. There is a gain of around 29% (78% vs 49%) in terms of probability to Go using the proposed design and the chance to stop the development is much lower (18%) with this design compared to the classical approach. It can also be observed that under this scenario PHR_2$_{q_2}$ every time we continue in the subpopulation then at the end of the trial we proceed to the drug development (probability to Go is 100% when we continue in the subpopulation).

Under PHR_2$_{q_1}$ and PHR_2$_{q_2}$ scenarios, it can be observed that there is a higher chance of declaring the presence of a cutoff (61% and 78% for scenario HP_2$_{q_1}$ and HP_2$_{q_2}$ respectively) and the mean estimates of the cutoff value are quite close to the true cutoffs.

Under the SPHR$_{q_2}$ scenario, it can be observed that there is a lower chance (12% instead of 24%) to stop for futility at the interim analysis and a higher chance to proceed with the full population (60% compared to 47%) compared to the PHR$_{q_2}$ scenario. In terms of overall probabilities at the final analysis, it can be observed that there is a higher chance of Go (73% instead of 58%) considering the biomarker-guided design and similar probability of No Go compared to the PHR$_{q_2}$ scenario. In addition, under this specific scenario it can be observed that at the end of the trial, there is no chance of being in the consider zone. Finally, under this additional SPHR$_{q_2}$ scenario, there is 53% chance of declaring the presence of a cutoff instead of 42% (under scenario PHR$_{q_2}$). In terms of comparison of probabilities in the decision-making with the classical design, the same patterns as for scenario PHR$_{q_2}$ can be observed here.

Scenario	p_0	p_1	q_+	Overall p	TV/LRV	Patients at IA
NPMR_2	19%	19%	50%	19%	30%/19%	14
NPLR_2	0%	0%	50%	0%	30%/19%	14
PHR_2$_{q_1}$	19%	41%	50%	30%	30%/19%	14
PHR_2$_{q_2}$	10%	60%	30%	25%	30%/19%	14
SPHR$_{q_2}$	2.5%	27.5%	30%	10%	15%/5%	20

TABLE 1 True response rates in the BMK- subgroup (p_0) and in the BMK+ subgroup (p_1). The prevalence of BMK-positive patients is indicated by q_+ and the overall response rate in the full population is p. TV and LRV are used at the FA.

Scenario	Probability of declaring a cutoff	ESS(25th, 75th percentiles)	E[cutoff](25th, 75th percentiles)
NPMR_2	0.28	18 (15, 22)	3.44 (2.68, 4.22)
NPLR_2	0	-	-
PHR_2$_{q_1}$	0.61	22 (17, 27)	3.67 (3.09, 4.33)
PHR_2$_{q_2}$	0.78	20 (16, 27)	4.08 (3.86, 4.48)
SPHR$_{q_2}$	0.53	25 (16 , 33)	4.27 (3.93, 4.8)

TABLE 2 Probability to find a cutoff, Expected Sample Sizes (ESS) and average value for the biomarker cutoff (E[cutoff]) and its 25th and 75th percentiles for additional scenario.

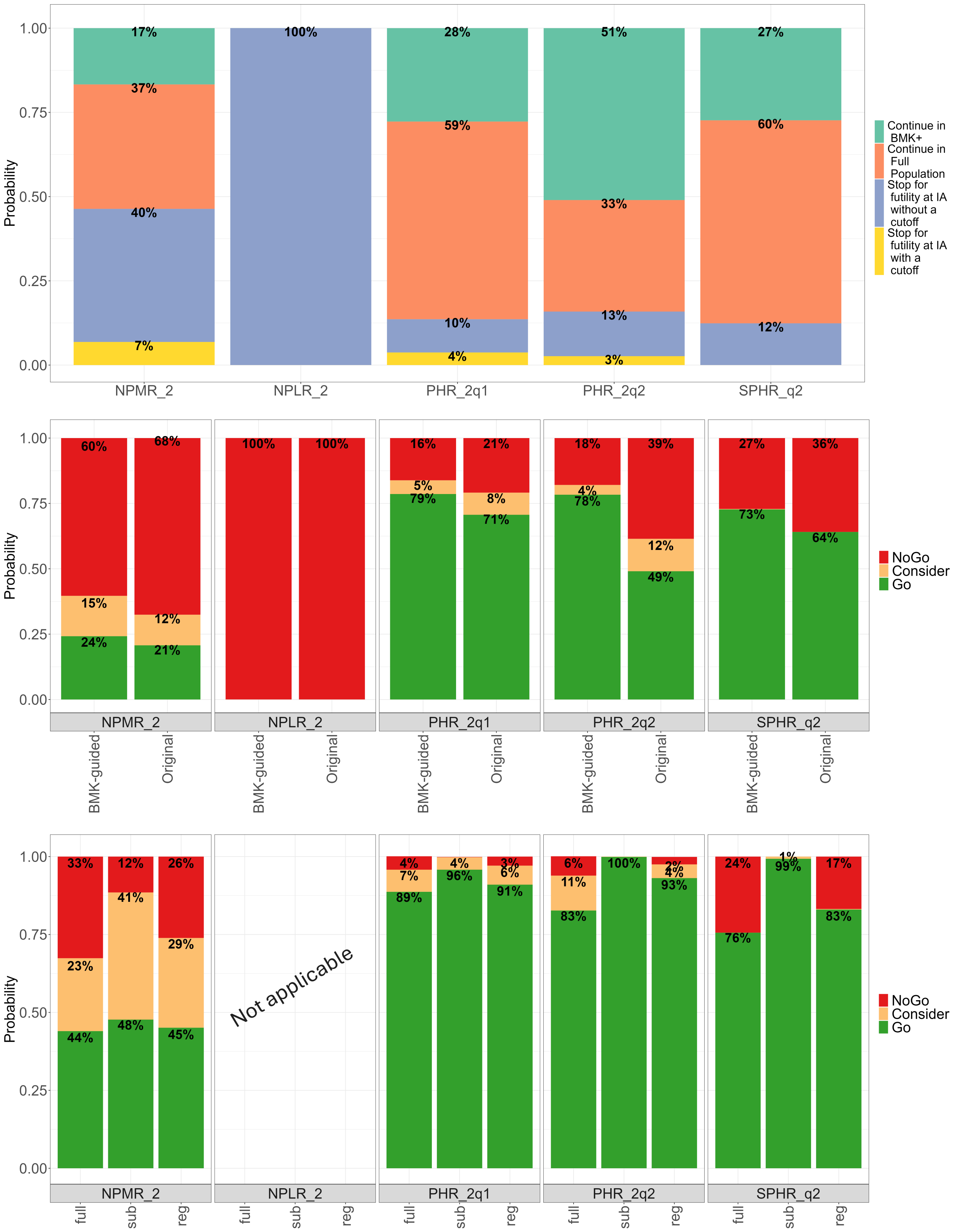

FIGURE 5 Top Panel: decisions at the IA for the proposed design. Middle Panel: Overall decisions at the end of the trial for both designs regardless of the population and the timing of the analysis for all scenarios. Bottom Panel: conditional decisions at the end of the trial for the proposed design with full or sub-population or regardless of the population conditional on continuing to the second stage for all scenarios.

3.2 | Different biomarker distributions

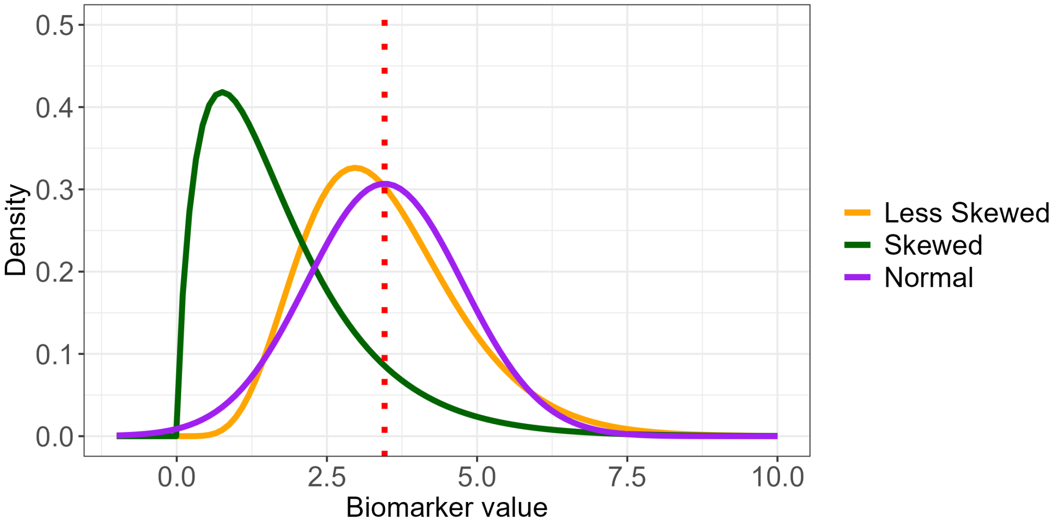

FIGURE 6 Distributions of the biomarker. The 'Normal' distribution is the one considered in the main analysis. The 'Less Skewed' gamma distribution has the same mean as the 'Normal' distribution and the 'Skewed' gamma distribution has the same variance as the 'Normal' distribution. The red vertical line corresponds to $\mu_X = 3.46$.

3.2.1 | Less Skewed Gamma distribution

Scenario	Probability of declaring a cutoff	ESS (25th, 75th percentiles)	E[cutoff](25th, 75th percentiles)
$NPHR_{q_1}$	0.24	22 (17, 27)	3.53 (2.71, 4.21)
$NPHR_{q_2}$	0.24	22 (17, 27)	3.48 (2.67, 4.15)
$NPMR_{q_1}$	0.09	18 (15, 19)	3.6 (3.04, 4.13)
$NPMR_{q_2}$	0.09	18 (15, 20)	3.64 (3.1, 4.17)
$NPLR_{q_1}$	0.00	-	-
$NPLR_{q_2}$	0.00	-	-
PHR_{q_1}	0.49	22 (17, 27)	3.88 (3.32, 4.4)
PHR_{q_2}	0.42	20 (15, 27)	4.2 (3.72, 4.68)
PMR_{q_1}	0.30	21 (16, 27)	3.82 (3.24, 4.38)

TABLE 3 Probability to find a cutoff, Expected Sample Sizes (ESS) with its 25th and 75th percentiles and average value for the biomarker cutoff (E[cutoff]) with its 25th and 75th percentiles for all scenarios. The distribution of the biomarker is 'Less Skewed'.

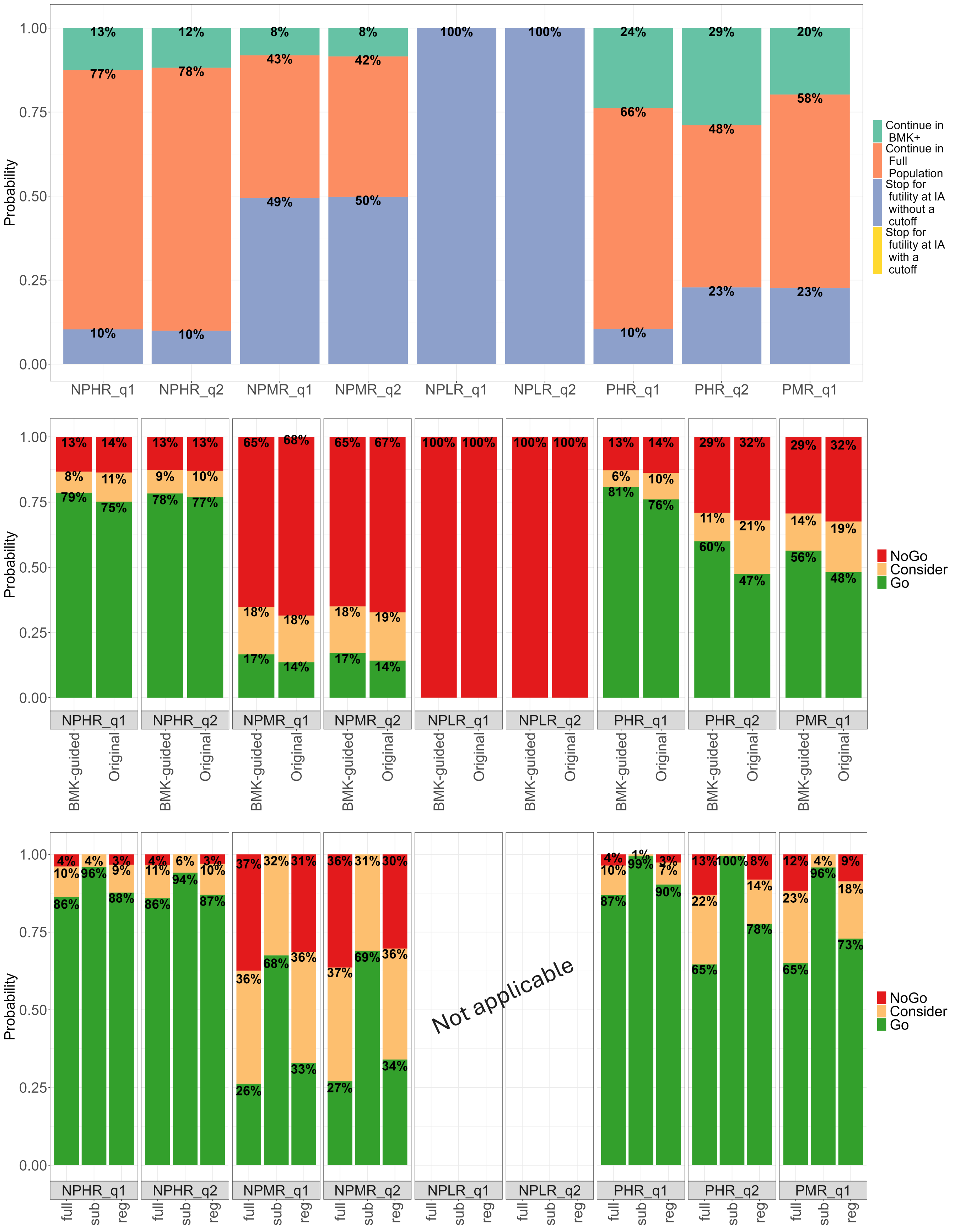

FIGURE 7 Top Panel: decisions at the IA for the proposed design. Middle Panel: Overall decisions at the end of the trial for both designs regardless of the population and the timing of the analysis for all scenarios. Bottom Panel: conditional decisions at the end of the trial for the proposed design with full or sub-population or regardless of the population conditional on continuing to the second stage for all scenarios. The distribution of the biomarker is 'Less Skewed'.

3.2.2 | Skewed Gamma distribution

Scenario	Probability of declaring a cutoff	ESS (25th, 75th percentiles)	E[cutoff](25th, 75th percentiles)
$NPHR_{q_1}$	0.25	22 (16, 27)	1.83 (0.94, 2.47)
$NPHR_{q_2}$	0.24	22 (16, 27)	1.8 (0.95, 2.43)
$NPMR_{q_1}$	0.09	18 (15, 20)	2.06 (1.35, 2.65)
$NPMR_{q_2}$	0.08	18 (15, 20)	2.03 (1.33, 2.61)
$NPLR_{q_1}$	0.00	-	-
$NPLR_{q_2}$	0.00	-	-
PHR_{q_1}	0.49	22 (17, 27)	2.16 (1.47, 2.69)
PHR_{q_2}	0.43	19 (15, 27)	2.51 (1.99, 2.97)
PMR_{q_1}	0.31	20 (16, 27)	2.16 (1.48, 2.71)

TABLE 4 Probability to find a cutoff, Expected Sample Sizes (ESS) with its 25th and 75th percentiles and average value for the biomarker cutoff (E[cutoff]) with its 25th and 75th percentiles for all scenarios. The distribution of the biomarker is ‘Skewed’.

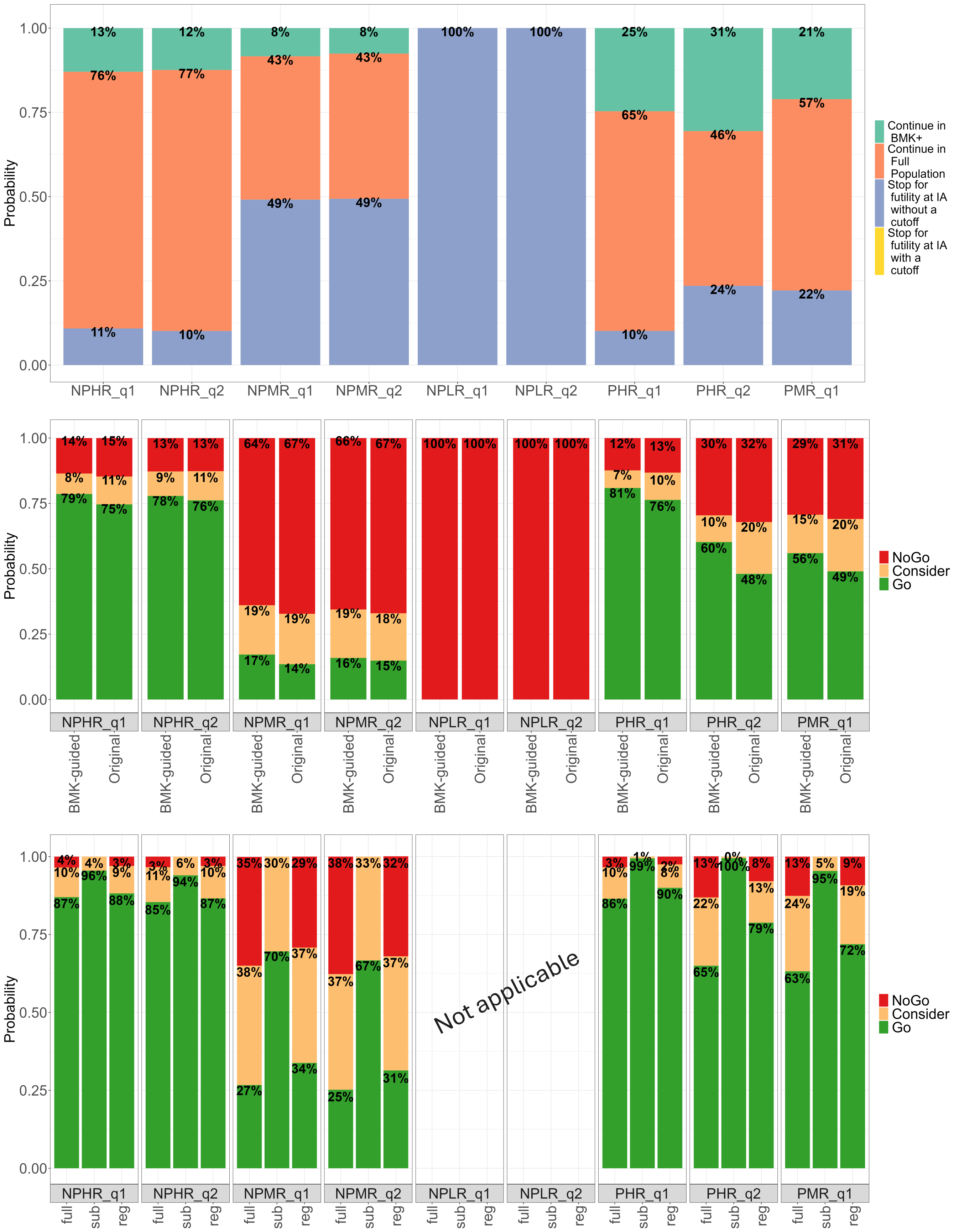

FIGURE 8 Top Panel: decisions at the IA for the proposed design. Middle Panel: Overall decisions at the end of the trial for both designs regardless of the population and the timing of the analysis for all scenarios. Bottom Panel: conditional decisions at the end of the trial for the proposed design with full or sub-population or regardless of the population conditional on continuing to the second stage for all scenarios. The distribution of the biomarker is ‘Skewed’.

3.3 | Prevalence of 15%

Scenario	p_0	p_1	Prevalence q_+	Overall p
NPHR$_{q_3}$	15%	15%	15%	15%
NPMR$_{q_3}$	5%	5%	15%	5%
NPLR$_{q_3}$	0%	0%	15%	0%
PHR$_{q_3}$	5%	25%	15%	8%
PHR_2$_{q_3}$	2.5%	27.5%	15%	6.25%
PMR$_{q_3}$	5%	15%	15%	6.5%

TABLE 5 True response rates in the BMK- subgroup (p_0) and in the BMK+ subgroup (p_1). The prevalence of BMK-positive patients is indicated by q_+ and the overall response rate in the full population is p.

Scenario	Probability of declaring a cutoff	ESS (25th, 75th percentiles)	E[cutoff](25th, 75th percentiles)
NPHR$_{q_3}$	0.25	23 (17, 27)	3.45 (2.73, 4.15)
NPMR$_{q_3}$	0.09	18 (15, 20)	3.44 (2.94, 3.94)
NPLR$_{q_3}$	0.00	-	-
PHR$_{q_3}$	0.26	19 (15, 21)	3.9 (3.12, 4.81)
PHR_2$_{q_3}$	0.25	18 (15, 17)	4.05 (3.25, 4.92)
PMR$_{q_3}$	0.19	18 (15, 20)	3.71 (3.02, 4.52)

TABLE 6 Probability to find a cutoff, Expected Sample Sizes (ESS) with its 25th and 75th percentiles and average value for the biomarker cutoff (E[cutoff]) with its 25th and 75th percentiles for all scenarios. The distribution of the biomarker is is 'Normal' and the prevalence (q_+) is 15%.

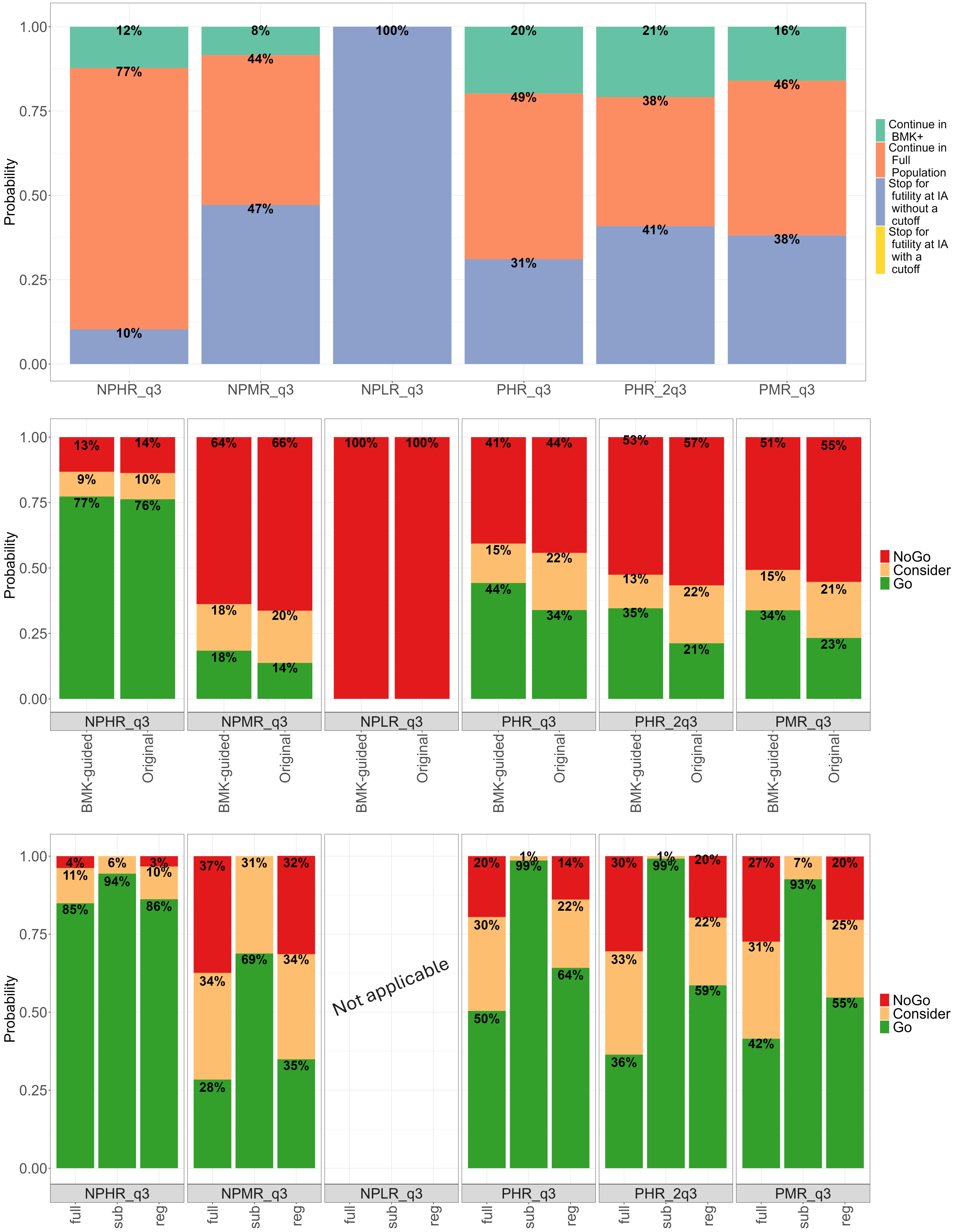

FIGURE 9 Top Panel: decisions at the IA for the proposed design. Middle Panel: Overall decisions at the end of the trial for both designs regardless of the population and the timing of the analysis for all scenarios. Bottom Panel: conditional decisions at the end of the trial for the proposed design with full or sub-population or regardless of the population conditional on continuing to the second stage for all scenarios. The distribution of the biomarker is 'Normal' and the prevalence (q_+) is 15%.

3.4 Interim analysis done after 18 patients have been treated

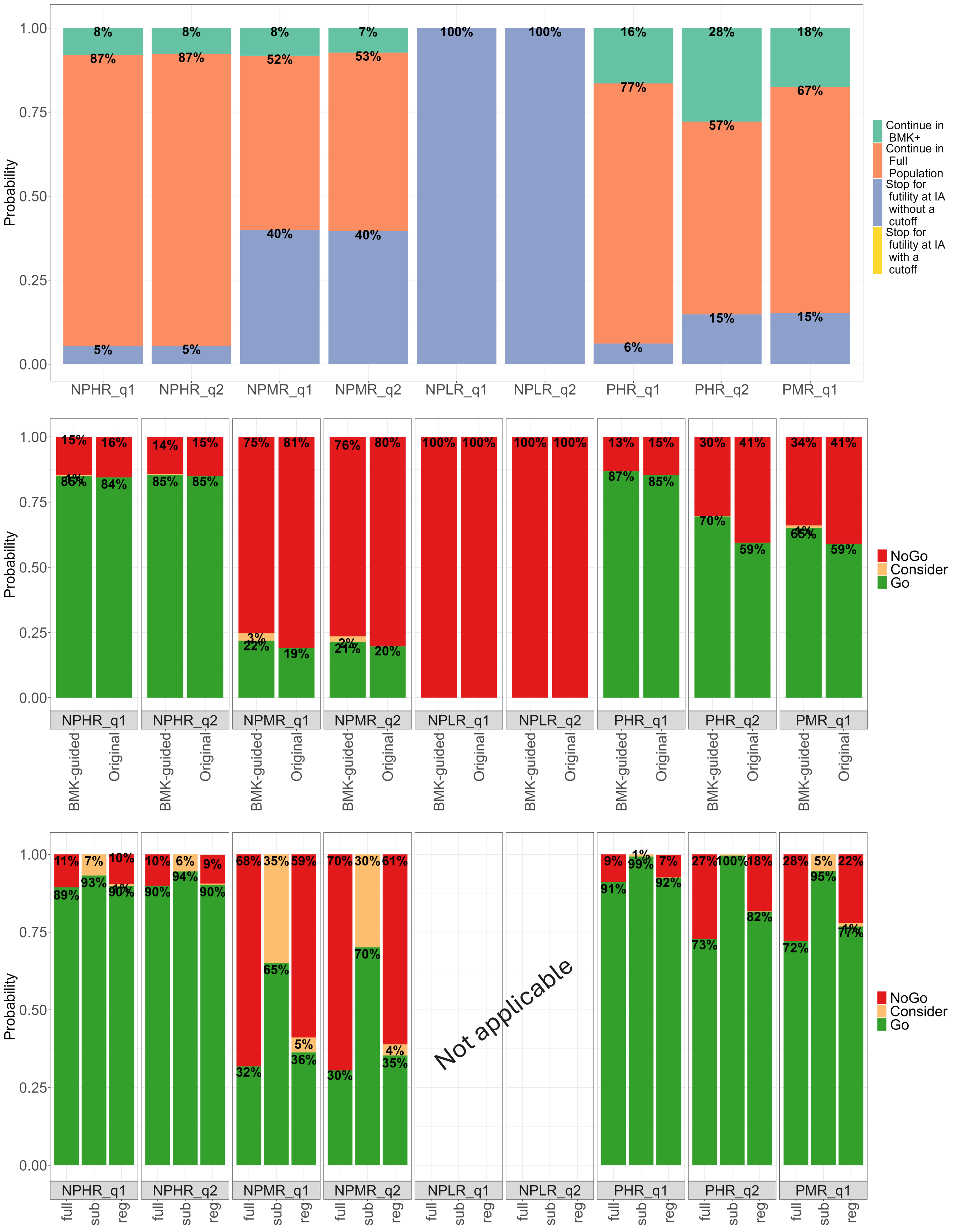

FIGURE 10 Top Panel: decisions at the IA for the proposed design. Middle Panel: Overall decisions at the end of the trial for both designs regardless of the population and the timing of the analysis for all scenarios. Bottom Panel: conditional decisions at the end of the trial for the proposed design with full or sub-population or regardless of the population conditional on continuing to the second stage for all scenarios. The distribution of the biomarker is ‘Normal’ and the number of patients at the interim analysis is 18.

3.5 | Additional metric

The table 7 describes the % of simulations in which the biomarker criterion is not satisfied at the IA but then is satisfied at the FA, regardless of continuing in the full or restricted biomarker-positive population (overall) or conditioning on continuing in the full population at the IA (conditional).

p_0	p_1	Patients at IA	Conditional	Overall
15%	15%	14	16%	2%
15%	15%	14	16%	2%
5%	5%	14	3%	1%
5%	5%	14	3%	1%
0%	0%	14	0%	0%
0%	0%	14	0%	0%
5%	25%	14	38%	6%
2.5%	27.5%	14	26%	6%
5%	15%	14	18%	3%
15%	15%	18	17%	1%
15%	15%	18	18%	2%
5%	5%	18	2%	0%
5%	5%	18	3%	0%
0%	0%	18	0%	0%
0%	0%	18	0%	0%
5%	25%	18	48%	4%
2.5%	27.5%	18	31%	4%
5%	15%	18	22%	2%

TABLE 7 True response rates in the BMK- subgroup (p_0) and in the BMK+ subgroup (p_1). The table describes the % of simulations in which the biomarker criterion is not satisfied at the IA but then is satisfied at the FA, regardless of continuing in the full or restricted biomarker-positive population (overall) or conditioning on continuing in the full population at the IA (conditional).

ACKNOWLEDGMENTS

This work was supported by Institut de Recherches Internationales Servier. The results reported herein are part of a collaboration between Servier, Saryga, and P Mozgunov and A Serra, whose research is supported by the National Institute for Health and Care Research (NIHR Advanced Fellowship, Dr Pavel Mozgunov, NIHR300576). The views expressed in this publication are those of the authors and not necessarily those of the NHS, the National Institute for Health and Care Research or the Department of Health and Social Care (DHCS). P Mozgunov and A Serra received funding from UK Medical Research Council (MC UU 00040/03). For the purpose of open access, the author has applied a Creative Commons Attribution (CC BY) license to any author accepted manuscript version arising

Author contributions

All authors have directly participated in the planning and execution of the presented work.

Financial disclosure

None reported.

Conflict of interest

JG and SG are the employees of Institut de Recherches Internationales Servier. GSH is President of Saryga SAS. PM and AS served as statistical consultants for Institut de Recherches Internationales Servier and Saryga SAS.

SUPPORTING INFORMATION

Additional supporting information may be found online in the Supporting Information section at the end of this article.
